# Mental imagery and stress: the mediating role of self-efficacy in competitive martial arts athletes

**DOI:** 10.3389/fpsyg.2025.1517718

**Published:** 2025-02-18

**Authors:** Donatella Di Corrado, Patrizia Tortella, Marinella Coco, Maria Guarnera, Matej Tusak, Maria Chiara Parisi

**Affiliations:** ^1^Department of Sport Sciences, Kore University, Enna, Italy; ^2^Department of Biomedical and Biotechnological Sciences, University of Catania, Catania, Italy; ^3^Department of Psychology, Kore University, Enna, Italy; ^4^Department of Social and Humanistic Sciences in Sport, Faculty of Sport, University of Ljubljana, Ljubljana, Slovenia

**Keywords:** mental imagery, martial arts, performance, stress, competition

## Abstract

**Introduction:**

Martial arts are diverse systems of combat practices, each with its unique techniques, philosophies, and histories. Mental imagery is a multisensory experience that integrates various senses to create vivid mental representations, evoking the physical attributes of people, places, or objects not currently within our perception. In sports contexts, studies have indicated that martial arts often use imagery to enhance performance.

**Methods:**

This study aimed to investigate the mediating role of self-efficacy in the relationship between static (visualizing still scenes or concepts) and dynamic (focusing on motion and action) vividness, controllability (manipulating the imagery experience), and the degree of stress, in a group of 110 martial arts athletes (61 males and 49 females), aged between 21 and 23 years old (*M* = 22.47, *SD* = 0.75). Participants were competitive athletes of karate, taekwondo and judo, having a minimum of 13 years of training skill in the sport. They completed the Vividness of Visual Imagery Questionnaire, the Vividness of Movement Imagery Questionnaire-2, the Mental Image Transformation Task, The General Self-Efficacy Scale, and Measuring Psychological Stress Test.

**Results:**

A 2 × 3 (gender × sport type) between groups MANOVA showed significant differences in imagery dimensions by sport type. The results of the mediation analysis showed that self-efficacy plays a mediating role between imagery and stress.

**Discussion:**

The study advanced exploration in the field of mental imagery training, providing additional evidence for its importance in improving performance and managing stress in athletes.

## Introduction

Martial arts encompass a variety of combat practices, and can be broadly categorized into traditional styles (like Karate, Taekwondo, and Judo), modern self-defense systems (like Krav Maga), and mixed martial arts. Each style has its own techniques, philosophies, and training methods. However, they share several general characteristics that define their essence and purpose. At their core, martial arts emphasize discipline, respect, physical fitness, and self-defense (Gavagan and Sayers, 2017; Barnamehei et al., 2020). Practitioners not only learn physical skills but also cultivate mental strength, confidence, skill of visualization, and adaptability to stressors. Specifically, they often use visualization techniques to complement their physical training, creating a powerful synergy between the mind and body, enhancing performance, and skill acquisition (Lindsay et al., 2023). Mental imagery is a multisensory experience that integrates various senses to create vivid mental representations, evoking the physical attributes of people, places, or objects not currently within our perception. It is crucial for executing movements and plays a significant role in overall human functioning (Commodari et al., 2024). In sports contexts, imagery is described as a state where individuals visualize themselves, third or first person, performing skills necessary for upcoming tasks or enhancing their performance (Cumming et al., 2023). This imagery can arise from both conscious and unconscious recall processes, allowing a person to “see” an image or “experience” a movement in their mind without actually experiencing it in reality. Imagery is crucial in this context, as it enhances performance in motor tasks. By mentally rehearsing movements, athletes can refine their skills, boost confidence, and improve overall execution (Simonsmeier et al., 2021). In mental imagery, dynamic (the body’s perceptual involvement during movement and action) and static (the visual fixed aspects of what a person sees) are the most commonly used sensory methods for creating mental images (Moran and O’Shea, 2020). These approaches help individuals enhance their performance by integrating bodily sensations and kinaesthetic actions with visual representations (Guillot et al., 2024). Imagery is indeed a complex construct. Several studies have identified a link between high athletic performance and proficiency in mental rotation. They also highlight a strong connection between motor practice and imagery ability, which is connected to mental rotation processes (Voyer and Jansen, 2017; Geisen et al., 2024; Di Corrado et al., 2020).

The skill of generating vivid mental images differs from the ability to control and manipulate those images. In this context, cognitive and sports psychology have developed tools to differentiate mental imagery ability into two components: controllability, defined as the ability to mentally manipulate an image accurately, and vividness, which refers to the sensory richness of an image (Cumming and Eaves, 2018).

Vividness is typically assessed through self-report questionnaires that capture individuals’ subjective perceptions of static and dynamic images quality, which may not reflect the actual quality of the imagery (Bedir and Erhan, 2021). In contrast, controllability can be evaluated using objective criteria, such as mental rotation tasks that require cognitive manipulation and spatial transformation of imagined objects (Saimpont et al., 2015). Sport psychology presents mixed findings regarding the relationship between the two components of imagery - vividness and controllability - and their impact on athletic performance. An individual may excel in one type of imagery task while showing lower abilities or none in others. This variability underscores the nuanced nature of imagery skills, where strengths can differ across different tasks or contexts (Smith and Wakefield, 2015). In martial arts, vivid imagery can enhance performance by allowing practitioners to visualize techniques and scenarios effectively; on the contrary, the ability to manipulate mental images of objects is crucial for spatial awareness, movement planning, and executing techniques (Munroe-Chandler and Guerrero, 2018).

Some studies indicate that individuals with high vividness, as assessed by self-report methods, learn new movements more quickly than those with lower vividness abilities. Furthermore, these individuals tend to perform movements with greater accuracy (Parnabas et al., 2015; Cunanan et al., 2018).

Conversely, other research shows that superior performance among athletes is linked to controllability rather than vividness. For example, Moreau et al. (2010), found no significant differences in vividness across varying levels of expertise in fencing, judo, and wrestling, while controllability showed positive effects. In contrast, Di Corrado et al. (2014), reported that dancers and karate practitioners scored higher in both vividness and controllability compared to a control group of non-athletes. This highlights the complexity of the relationship between these components and athletic performance. Additionally, research has highlighted that mental imagery not only improves sports performance and outcomes during actual competitions, but also helps reduce pre-match stressful symptoms. Stress is the feeling of being overwhelmed by mental or emotional pressure. It is also a significant factor that can adversely affect an individual’s cognitive and physical capabilities (Budnik-Przybylska et al., 2023; Di Corrado et al., 2021). Nevertheless, it is an inevitable part of life and sport, characterized as a physical, mental, or emotional demand that disrupts the body’s homeostasis. While a certain level of stress can be beneficial - providing the pressure and demands needed to enhance performance - excessive stress can be detrimental.

Stress can manifest as acute, episodic, or chronic, with episodic stress being the most common. If acute stress is not managed effectively, it can lead to long-term negative effects on performance (Di Corrado et al., 2021; Wearne et al., 2019).

When athletes perceive stress as unmanageable, they often experience a decline in performance levels, which can range from mild to severe (Cunanan et al., 2018). Interestingly, many studies have recognized that maintaining positive functioning can be a normative response even in the face of emotional distress. This refers to an adaptive process where people use cognitive efforts to cope with adversity, leading to well-being and adaptability to stressors (Kellmann et al., 2018). Self-efficacy is seen as an essential resource and a key factor for success, emphasizing its role as a protective element or mediator that fosters more adaptive responses to stress and adversity. It refers to an individual’s belief in their capacity to utilize motivational, and cognitive resources to address situational demands (Di Corrado et al., 2022). Research has confirmed its significant role in adaptive coping styles during stressful situations, showing that individuals with high self-efficacy tend to have greater control over their thoughts and exhibit more stability in goal attainment (Peers et al., 2020; Popovych et al., 2020; Di Corrado et al., 2023). Referring to mental visualization techniques used in sports, in the current study, we have explored traditional styles of martial arts, including judo, karate, and taekwondo. Each discipline incorporates different elements that can influence the effectiveness of imagery. At this regard, studies have indicated that judoka often use imagery to enhance performance in techniques, timing, and situational awareness; karate practitioners frequently use imagery to visualize complex movements and technique precision; taekwondo athletes often employ imagery to visualize competitive scenarios and dynamic movements (Piedade et al., 2022; Russo and Ottoboni, 2019: Beheshti et al., 2021).

The choice of imagery techniques may depend on the specific skills and strategies emphasized in each discipline. The relationship between mental imagery, self-efficacy, and stress, respectively, in judo, karate, and taekwondo has been a focus of various studies, revealing interesting insights into how imagery can influence athletes’ mental states and performance. Research indicated that imagery can significantly enhance self-efficacy in karate athletes (Simonsmeier et al., 2021; Khodabandelou and Salehian, 2023). By visualizing successful performances, athletes can boost their confidence, leading to improved execution of techniques during competitions and also managed stress. Di Corrado et al. (2019), revealed that both vividness and controllability in imagery significantly predicted self-efficacy. In judo, athletes who engaged in imagery reported higher levels of self-efficacy, making athletes feel more prepared for matches and more resilient (Andreato et al., 2022). A study by Silva et al. (2018), found that elite judoka used imagery to enhance their mental preparation before matches. They reported using imagery to visualize specific techniques and scenarios, which helped improve their confidence and performance under pressure.

Julvanichpong et al. (2022), have shown that vivid mental rehearsal of techniques and sparring scenarios boosted confidence in taekwondo athletes, feeling more capable and focused during competitions.

Despite the large amount of research in the strong positive relationship between imagery and self-efficacy conducted separately in all three sports, very little attention has been devoted to the precise relationship between each dimension of imagery vividness (static and dynamic), controllability (i.e., the athlete’s ability to manipulate and direct their mental imagery), and stress levels. Given the limited research, the purpose of this study was to test the mediating role of self-efficacy in the association between Static Imagery, External dynamic Imagery, and Internal dynamic Imagery, Controllability, and the degree of Stress among judo, karate, and taekwondo athletes.

More specifically, we hypothesized the following:

*H1: There are significant relationships among vividness, controllability, and stress levels, across different types of martial arts.*

*H2: Self-efficacy mediates the relationship between each dimension of imagery and stress.*

**Figure 1:**
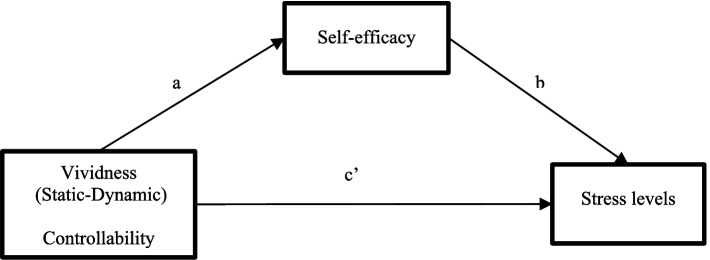
Hypothesized mediation model explaining the potential mediating effect of self-efficacy on the relationship between each of dimension of imagery vividness and controllability, and stress levels.

In our hypothesized model (Figure 1), the total effects included a direct effect pathway (path c’) of each dimension of imagery vividness (Static Imagery, External dynamic Imagery, and Internal dynamic Imagery), and Controllability on the stress levels, and a total indirect pathway (mediated: path ab) of each level of imagery vividness and controllability, on the stress levels through self-efficacy.

## Materials and methods

### Participants and procedure

Group sample sizes were decided by power analysis, with G*Power being used in a one-way between-subjects ANOVA (Faul et al., 2007). The input parameters were effect size (*ŋ*^2^ = 0.70), alpha level of 0.05, power (0.95%) and the number of groups sampled (three groups). Finally, the result revealed the need for 36 participants in each of the three groups (108 in all). Therefore, a total of *n* = 110 participants (61 males and 49 females), aged between 21 and 23 years old (*M* = 22.47, *SD* = 0.75), were recruited to take part in the research. The athletes were recruited according to the style they practiced: taekwondo (*n* = 36), karate (*n* = 34) and judo (*n* = 40) group. They were active competitors and had a minimum of 13 years of training skill in the sport (*M* = 15.05, *SD* = 1.18). They usually trained five or six times per week (*M* = 9.6 h, *SD* = 1.3). The inclusion criteria for participants were: (a) at least 18 years old; (b) availability for the entire data collection period; (c) no motor or neurological deficits; and (d) ability to provide informed consent. Participants were excluded if they did not meet these criteria. Prior to the study, all participants received a comprehensive explanation of the protocols and signed an informed consent form for all tests. The procedures adhered to the ethical guidelines of the Declaration of Helsinki, and ethical approval was granted by the University Enna Kore Internal Review Board for research (UKE-IRBPSY-09.23.11). Measurements were conducted individually in groups of four participants, in a secluded area near the training facilities, and not immediately before or after competitions to reduce external distractions. All participants completed a series of tests under the supervision of two researchers, with confidentiality of their responses guaranteed. The descriptive characteristics of the participants are shown in Table 1.

**Table 1:** Descriptive statistics of the sample.

	Gender M ± SD	Age M ± SD (range)	Training skill years M ± SD (range)
Taekwondo (*n* = 36)	15 m; 21 f	22.50 ± 0.74 (21–23)	15.19 ± 1.12 (13–16)
Karate (*n* = 34)	18 m; 16 f	22.37 ± 0.79 (20–23)	14.85 ± 1.30 (13–16)
Judo (*n* = 40)	28 m; 12 f	22.45 ± 0.75 (21–23)	15.02 ± 1.12 (14–16)

### Measures

#### Imagery vividness assessment

##### Static imagery

The Vividness of Visual Imagery Questionnaire (Marks, 1973) in the Italian adapted version (Antonietti and Crespi, 1995) was used to assess the participant’s Static imagery. The instrument is a 16-item self-report tool designed to assess the vividness of visual mental imagery across four subscales, with four items dedicated to each. Participants are prompted to visualize scenes, such as characteristics of a friend or parent, the climate, or a country, and then rate the clarity and vividness of these images on a five-point scale: 1: “*No image at all (only a sense of knowing the object)*”; 2: “*Vague and dim*”; 3: “*Moderately clear and vivid*”; 4: “*Clear and fairly vivid*”; and 5: “*Perfectly clear and vivid, just like normal perception*.” Higher scores reflect greater vividness. The instrument has demonstrated a mean Cronbach’s *α* of 0.89, indicating strong internal consistency (McKelvie, 1995). The internal consistency for the current study is good (α = 0.86).

##### Dynamic imagery

The Vividness of Movement Imagery Questionnaire-2 (Roberts et al., 2008) is a self-report questionnaire for measuring Dynamic imagery and includes 12 items. Participants evaluate their ability to visualize or kinaesthetically imagine a movement using a 5-point Likert scale, where 1 indicates “*perfectly clear and vivid*” and 5 indicates “*no image at all, only a sense of knowing you are ‘thinking’ of the skill*.” The instrument assesses three distinct types of movement imagery: 1. External dynamic imagery, which refers to the visualization of movement from an outsider’s perspective; 2. Internal dynamic imagery, which involves visualizing oneself performing the movement; 3. Kinaesthetic dynamic imagery, which captures the somatosensory experience of executing the task, as if one were truly engaged in the activity. The tool has demonstrated a Cronbach’s α of 0.91, indicating excellent internal consistency. The internal consistency for the current study is very good (α = 0.88). To obtain the prevalence of different types of viewpoint imagination, our current study focused on two kinds of movement imagery: external viewpoint and internal viewpoint.

#### Imagery controllability assessment

##### Subtraction of parts

**Figure 2:**
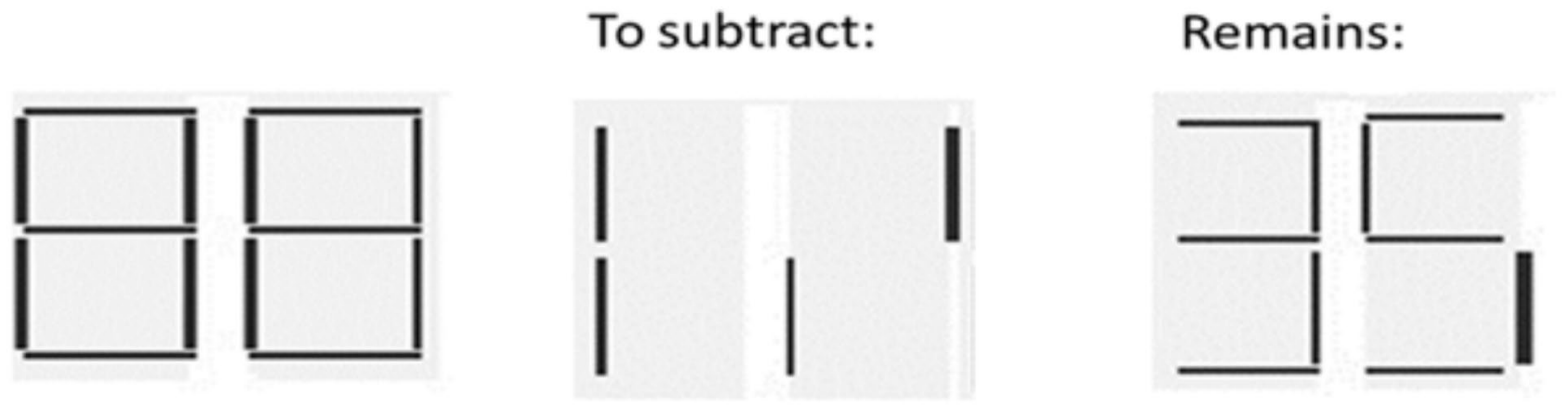
Mental image transformation task subtraction of parts.

It consists of mentally taking away a part of an image in order to compose or decompose complete figures. This task was extracted from Mental Imagery Test (Di Nuovo et al., 2014). Participants were shown two 17x12cm cards representing a full-lighted digital clock on the first card, while the second card contained the part(s) of the picture that needed to be taken away (Figure 2). Participants were asked to image complete clock without the subtracting parts and name the resulting image. Specifically, participants received the following verbal instructions: “Look at this (complete clock) without this (subtracting parts). What does this become?” Participants received three trials and were tested individually in a single session lasting about 10 min. The subtest has reported an alpha coefficient of *r* = 0.68.

#### Stress levels

The level of stress was assessed using the Italian version (Di Nuovo et al., 2000) of the Psychological Stress Measure (PSM – Lemyre et al., 1990), a self-administered questionnaire consisting of 49 items. It is based on different aspects related to the perception that the individual has of its condition. The choice of responses is made of a scale (Likert-type) whose possible answers are 1–4 (from “*null*” to “*much*”). A total score of stress level can be found by sum of all scores. The internal consistency measured for our data was good, showing a *α* > 0.89.

#### Self-efficacy

The Italian version of the General Self Efficacy Scale (Schwarzer and Jerusalem, 1995; Di Nuovo and Magnano, 2013) is a 10-item scale designed to assess optimistic self-beliefs to cope with demands in life and reach goals (e.g., “Even if someone opposes, I can find the means and strength to achieve what I desire”; “I am confident that I can cope adequately with unexpected events”). Response options range on a 5-point Likert format from (5) *Completely agree to* (1) *Completely disagree*. Higher scores indicate a stronger sense of self-efficacy. In the present sample, Cronbach’s α was 0.89.

#### Statistical analyses

Data were expressed as means (*M*) ± standard deviations (*SD*) and the range. A 2 × 3 (gender × sport type) multivariate analysis of variance (MANOVA) was conducted to compare the mean in main effects and interactions of gender (male and female) and sport type (taekwondo/karate/judo) on the data of the study variables. Follow-up analysis of variance (ANOVA) and *t*-test with Bonferroni correction were then used to examine the significant differences. Gender was included as a control variable in the analysis to account for potential differences between male and female participants. Furthermore, Pearson’s correlation was used to determine the relationships between the selected variables. An a-priori power analysis was run with G*Power (Faul et al., 2007). To examine the significance of mediation, the recommendations put forth by Baron and Kenny (1986) were followed. Their Steps 1, 2 and 3 involve testing the significance of the relationship between the following: (1) the independent and the dependent variables (i.e., vividness/controllability and stress); (2) the independent and the mediator variables (i.e., vividness/controllability and self-efficacy); (3) the mediator and the dependent variables (i.e., self-efficacy and stress). If these steps are passed, one should determine in Step 4 whether the mediator variable reduces or eliminates the link between the independent variable and the dependent variable. For this purpose, a mediation analysis was conducted using Hayes (2013) PROCESS version 3.1 (Hayes PROCESS macro-Model 4 – Ohio, USA) computational tool for SPSS. This tool enables the estimation of path coefficients, standard errors, and different indexes of effect size, as well as the significance of the indirect effects obtained through the bootstrapping method with 5,000 repetitions, with a confidence interval (CI) of 95% (Preacher and Kelley, 2011). Statistical significance was set at *p* ≤ 0.05. Statistical analyses were processed using SPSS version 27.0 (IBM, Armonk, NY, USA).

## Results

Findings revealed significant multivariate effects only for sport type, Wilks *λ* = 0.002, *F* (12, 198) = 368.705, *p* = 0.001, *η*_p_^2^ = 0.957, power = 1.000. Means and standard deviations of study variables are reported in Table 2.

**Table 2:** Mean ± SD for the study variables.

		Taekwondo *n* = 36		Karate *n* = 34		Judo *n* = 40	
		Males *n* = 15	Females *n* = 21	Males *n* = 18	Females *n* = 16	Males *n* = 28	Females *n* = 12
Static imagery	M	42.60	44.33	76	76.87	71.29	70.92
	SD	1.54	3.26	2.42	1.54	1.15	0.79
External dynamic imagery	M	50.73	55.33	21.94	22.56	44.71	43.92
	SD	4.62	2.51	3.36	4.45	3.37	3.63
Internal dynamic imagery	M	51.13	53.48	24.22	22.19	44.32	46.08
	SD	6.54	5.07	3.71	4.79	3.13	4.35
Controllability	M	3.13	3.24	7.56	7.57	5.29	5.08
	SD	0.74	0.76	0.51	0.52	0.89	0.79
Self-efficacy	M	45.87	45.71	24.22	25.06	33.50	33.25
	SD	2.13	2.93	3.15	3.10	2.71	2.41
Stress	M	56.73	55.48	108	107.37	76	80.75
	SD	3.57	3.38	3.64	2.96	15.11	18.36

Follow up ANOVAs revealed significant univariate differences (*p* = 0.001) between sport type on all variable scores. Specifically, athletes of karate reported higher scores on Static Imagery: *F* (2, 107) = 2652.205, *p* = 0.001, η_p_^2^ = 0.981, power = 1,000 (taekwondo *M* = 43.61; karate *M* = 76.41; judo *M* = 71.18); Controllability: *F* (2, 107) = 303.230, *p* = 0.001, η_p_^2^ = 0.854, power = 1,000 (taekwondo *M* = 3.19; karate *M* = 7.56; judo *M* = 5.23); Stress: *F* (2, 107) = 224.849, *p* = 0.001, η_p_^2^ = 0.812, power = 1,000 (taekwondo *M* = 56; karate *M* = 107.71; judo *M* = 77.43), than athletes of other sports. Conversely, athletes of taekwondo reported higher scores on External dynamic Imagery: *F* (2, 107) = 654.865, *p* = 0.001, η_p_^2^ = 0.926, power = 1,000 (taekwondo *M* = 53.42; karate *M* = 22.24; judo *M* = 44.48); Internal dynamic Imagery: *F* (2, 107) = 377.573, *p* = 0.001, η_p_^2^ = 0.879, power = 1,000 (taekwondo *M* = 52.50; karate *M* = 23.26; judo *M* = 44.85); Self-efficacy: *F* (2, 107) = 498.140, *p* = 0.001, η_p_^2^ = 0.905, power = 1,000 (taekwondo *M* = 45.78; karate *M* = 24.62; judo *M* = 33.42), than athletes of other sports. Moreover, there was no significant difference (*p* > 0.05) across groups concerning gender.

Then, the relationships among variables were analysed with Pearson’s correlation (Table 3).

**Table 3:** Pearson’s correlation coefficients.

Variable	1	2	3	4	5	6
1. Static imagery	1					
2. External dynamic imagery	−0.762*	1				
3. Internal dynamic imagery	−0.740*	0.940*	1			
4. Controllability	0.833*	−0.881*	−0.840*	1		
5. Self-efficacy	−0.902*	0.853*	0.811*	−0.858*	1	
6. Stress	0.794*	−0.871*	−0.812*	0.839*	−0.850*	1

^*^ *p < 0.001.*

As can be observed, Dynamic Imagery (External/Internal) is positively associated with self-efficacy, and negatively associated with stress level. Conversely, Static Imagery and Controllability are negatively associated with self-efficacy, and positively associated with stress level. Lastly, self-efficacy is negatively associated with stress level.

Based on the correlation results, a mediation analysis was executed by entering the score of self-efficacy as mediator in the vividness/controllability and stress relationship. The mediation results are presented in Table 4, which contains the standardized *β*, indicating the intensity of the effect, and the 95% CIs, indicating the significance of the effect with a 5% probability of error (CIs that do not contain 0 are significant).

**Table 4:** Effects of imagery vividness on anger expression through resilience (standardized *β*).

Paths	Indirect effect			Direct effect		
	β	CI. 95%	*p*	β	CI. 95%	*p*
Static Imagery- > Self-efficacy- > Stress	1.037	0.721 ~ 1.351	<0.001	0.231	−0.130 ~ 0.594	0.20
External dynamic Imagery- > Self-efficacy- > Stress	−0.581	−0.804 ~ −0.367	<0.001	−0.918	−1.175 ~ −0.675	<0.001
Internal dynamic Imagery- > Self-efficacy- > Stress	−0.808	−1.038 ~ −0.593	<0.001	−0.636	−0.893 ~ −0.380	<0.001
Controllability- > Self-efficacy- > Stress	5.16	3.58 ~ 6.99	<0.001	5.06	3.06 ~ 6.85	<0.001

As can be observed, the results showed that Static Imagery had a significant indirect effect, mediated by self-efficacy, on stress levels (*β* = 1.037, SE = 0.02, *p* < 0.001). Thus, the relationship between Static imagery and stress was fully mediated by self-efficacy, with a decrement of stress levels. Furthermore, mediational analyses showed that External dynamic Imagery, Internal dynamic Imagery, and Controllability had both direct and indirect effects through self-efficacy on stress. Thus, these relationships were partially mediated by self-efficacy. The direct effect path-way of External dynamic Imagery (*β* = −0.918, SE = 0.02, *p* < 0.001) and Internal dynamic Imagery (*β* = −0.636, SE = 0.02, *p* < 0.001) had a significant and negative impact on stress levels, indicating Dynamic imagery as a significant predictor of stress.

## Discussion

Imagery plays a critical role in enhancing self-efficacy and managing stress in martial arts, contributing to athletes’ overall mental preparedness and performance. The aim of this study was to test the mediating role of self-efficacy in the association between Static Imagery, External dynamic Imagery, and Internal dynamic Imagery, Controllability, and the degree of Stress among judo, karate, and taekwondo athletes. We hypothesized that all the variables of imagery would be related to stress levels through the mediation of self-efficacy.

Data analysis partially confirmed the research hypotheses. In line with H1, findings showed that karate athletes reported significant higher scores on Static Imagery, Controllability and stress levels. On the other hand, results showed that taekwondo athletes reported significant higher scores on External dynamic imagery, Internal dynamic imagery, and self-efficacy.

According with our findings, Volgemute et al. (2023), found that athletes using dynamic imagery techniques reported higher self-efficacy and lower pre-competition anxiety compared to those using static imagery. This indicates that dynamic imagery may be more effective in high-pressure situations. Dynamic imagery plays a vital role in visualizing sparring scenarios and executing techniques effectively.

Research indicates that athletes who experience vivid dynamic imagery tend to feel more confident, as it enables them to rehearse and enhance their performance (Denis, 1985; Di Corrado et al., 2020). Moreover, it helps manage stress by preparing athletes for fast-paced situations, making them feel more capable of handling the unpredictability of competition (Di Corrado et al., 2014). Each sport employs a mix of both types of imagery, tailored to their specific skills and performance demands.

In judo, karate, and taekwondo, both static and dynamic imagery are utilized, but the emphasis varies depending on the nature of the sport (Stergiou et al., 2019). Specifically, karate practitioners primarily use static imagery to visualize the positions and postures required for katas (forms), which helps them understand stance, balance, and form. Dynamic imagery is employed when visualizing techniques in action (Franklin, 2022). Judoka use both static and dynamic imagery: static imagery to visualize grip positions and dynamic imagery to envision movements in response to their opponents. Lastly, static imagery is used less frequently by taekwondo athletes, who heavily rely on dynamic imagery to visualize high kicks, fast movements, and sparring scenarios. This approach helps them anticipate actions and enhance their performance under pressure (Fu et al., 2014; Patti et al., 2018).

Additionally, Pearson’s correlation analysis revealed very strong positive relationships between scores for self-efficacy and dynamic imagery variables (both external and internal), as well as a negative association with stress levels. In confirmation of such findings, research has shown that this positive correlation could likely be due to the fact that dynamic imagery can help athletes visualize themselves successfully completing a task, thereby increasing their self-confidence and belief in their ability to succeed.

Moreover, dynamic imagery can cultivate essential mental skills, including focus, concentration, and relaxation, which are vital for optimizing athletic performance (Lochbaum et al., 2023; Jekauc et al., 2023; Lochbaum et al., 2022).

Our findings also revealed a negative correlation between static imagery and controllability with self-efficacy, while positively linking them to stress levels. This can lead to a heightened stress response, both physically and mentally. Research supports this notion, indicating that focusing on negative or uncontrollable aspects of a situation through imagery can inadvertently exacerbate stress levels (Budnik-Przybylska et al., 2023; Zapała et al., 2021; Wearne et al., 2019). In other words, respondents who reveal higher physiological activation during imagery tasks are more likely to experience emotional instability, and stress. This could be explained using Yerkes and Dodson’s (1908) first law: with low worry, imagery did not play any role; however, when worry was high then imagery was moderate or high.

Lastly, a mediation analysis partially confirmed the research hypothesis (H2), highlighting that only the relationship between Static imagery and stress was fully mediated by self-efficacy, with a decrement of stress levels. Furthermore, mediational analyses showed that relationships between External dynamic Imagery, Internal dynamic Imagery, Controllability, and stress were partially mediated by self-efficacy. In line with our findings, previous research indicated that athletes who have higher self-efficacy use all imagery abilities more than athletes who have lower self-efficacy level (Volgemute et al., 2021; Mills et al., 2000). Specifically, athletes who feel confident in their abilities are better equipped to handle competition-related pressures, leading to improved focus and performance. Overall, our finding suggest that imagery serves multiple roles - enhancing skill acquisition, reducing anxiety, improving emotional regulation, and ultimately leading to better performance. These techniques are essential to mental preparation and contribute significantly to an athlete’s success in competitions.

A limitation of the present study could be that the cross-sectional nature of the work prevents any conclusions of causal relationships existing between variables. To establish causal relationships, future research could utilize a longitudinal design or prospective cohort studies. By following participants over time, researchers can track changes in variables and observe how one may influence the other. This would help establish causality by capturing temporal relationships between the variables.

Secondly, all data were obtained through self-reported questionnaires, which could present response bias. The participants might have underestimated or overestimated the relationship between the study variables.

To reduce response bias and to address potential inaccuracies in self-reports, researchers could employ multiple data collection methods or using different methods to verify the same information. For example, comparing self-reported data with other objective or third-party assessments. By applying these solutions, the study’s findings could be more robust and reliable, providing clearer insights into causal relationships and minimizing biases.

Future research could provide a more comprehensive understanding of how different types of imagery may specifically influence stress levels in athletes, with self-efficacy acting as a mediator. By examining these factors in greater detail, we could gain a more nuanced understanding of the interplay between mental strategies, stress management, and performance across diverse athletic settings.

## Conclusion

Athletes who believe in their skills are more likely to engage deeply with dynamic imagery, enhancing their performance and managing pre-performance stress. In conclusion, results demonstrated that vividness of imagery and controllability skills are correlated, while self-efficacy plays a crucial mediating role. Effective stress management through enhanced self-efficacy can lead to better mental performance in martial arts, highlighting the importance of psychological factors in physical training and competition. By enhancing self-efficacy through imagery strategies, coaches can create programs that focus on helping athletes manage challenges and reduce stress responses.

Moreover, by incorporating imagery strategies into regular training routines, coaches can help athletes manage stress more effectively, through imagery exercises where athletes visualize themselves succeeding in stressful situations, such as performing under pressure, recovering from mistakes, or staying focused during high-stakes moments.

## Data Availability

The original contributions presented in the study are included in the article/supplementary material, further inquiries can be directed to the corresponding author.
